# Growth Suppression of Colorectal Cancer by Plant-Derived Multiple mAb CO17-1A × BR55 via Inhibition of ERK1/2 Phosphorylation

**DOI:** 10.3390/ijms151121105

**Published:** 2014-11-14

**Authors:** Dong Hoon Kwak, Ghislain Moussavou, Ju Hyoung Lee, Sung Youn Heo, Kisung Ko, Kyung-A Hwang, Seung-Joo Jekal, Young-Kug Choo

**Affiliations:** 1 Institute of Glycoscience, Wonkwang University, Iksan, Jeonbuk 570-749, Korea; E-Mail: velvety7@nate.com; 2 Department of Biological Science, College of Natural Sciences, Wonkwang University, Iksan, Jeonbuk 570-749, Korea; E-Mails: strozzi37@gmail.com (G.M.); oooe64@naver.com (J.H.L.); akewhen@gmail.com (S.Y.H.); 3 Department of Medicine, Medical Research Institute, College of Medicine Chung-Ang University, Heukseok-ro 84, Seoul 156-756, Korea; E-Mail: ksko@cau.ac.kr; 4 Department of Agrofood Resources, National Academy of Agricultural Science, RDA, Suwon 441-853, Korea; E-Mail: kah366@korea.kr; 5 Department of Clinical Laboratory Science, Wonkwang Health Science University, Iksan 570-750, Korea; E-Mail: sjjei@wu.ac.kr

**Keywords:** anti-EpCAM, colon cancer, mAb^P^ CO17-1A × BR5, apoptosis, mAb^P^ CO17-1A, monoclonal antibody

## Abstract

We have generated the transgenic Tabaco plants expressing multiple monoclonal antibody (mAb) CO7-1A × BR55 by cross-pollinating with mAb CO17-1A and mAb BR55. We have demonstrated the anti-cancer effect of plant-derived multiple mAb CO17-1A × BR55. We find that co-treatment of colorectal mAbs (anti-epithelial cellular adhesion molecule (EpCAM), plant-derived monoclonal antibody (mAb^P^) CO17-1A and mAb^P^ CO17-1A × BR55) with RAW264.7 cells significantly inhibited the cell growth in SW620 cancer cells. In particular, multi mAb^P^ CO17-1A × BR55 significantly and efficiently suppressed the growth of SW620 cancer cells compared to another mAbs. Apoptotic death-positive cells were significantly increased in the mAb^P^ CO17-1A × BR55-treated. The mAb^P^ CO17-1A × BR55 treatment significantly decreased the expression of B-Cell lymphoma-2 (BCl-2), but the expression of Bcl-2-associated X protein (Bax), and cleaved caspase-3 were markedly increased. *In vivo*, the mAb^P^ CO17-1A × BR55 significantly and efficiently inhibited the growth of colon tumors compared to another mAbs. The apoptotic cell death and inhibition of pro-apoptotic proteins expression were highest by treatment with mAb^P^ CO17-1A × BR55. In addition, the mAb^P^ CO17-1A × BR55 significantly inhibited the extracellular signal-regulated kinase 1 and 2 (ERK1/2) phosphorylation in cancer cells and tumors. Therefore, this study results suggest that multiple mAb^P^ CO17-1A × BR55 has a significant effect on apoptosis-mediated anticancer by suppression of ERK1/2 phosphorylation in colon cancer compared to another mAbs. In light of these results, further clinical investigation should be conducted on mAb^P^ CO17-1A × BR55 to determine its possible chemopreventive and/or therapeutic efficacy against human colon cancer.

## 1. Introduction

Colorectal cancer also known as colon cancer or rectal cancer is the third most common cancer in men and the second most common cancer in women worldwide [1]. In recent years, the worldwide incidence rates of this cancer have been increasing steadily [2]. Despite advances in therapeutic interventions in recent decades, about 40% of patients will still eventually die because of disease mainly due to metastasis [3]. Although early stage colorectal cancer can be successfully treated surgically, advanced stage colorectal cancer frequently recurs and becomes fatal, even in patients receiving combination chemotherapy [4]. Previous studies revealed chemotherapeutic agents such as cisplatin are routinely used in the treatment of advanced stage colorectal cancer, but provide only minimal survival benefits, due to several factors, including drug resistance, side effects and toxicity [5,6].

Recently, development of cancer chemoprevention protocols employing natural or synthetic agent for the prevention or suppression to invasive cancer has been recognized as a field with enormous potential to reduce cancer burden [2,7]. Then, plants are being developed as a manufacturing platform for a range of pharmaceutical proteins such as vaccines, hormones and antibodies, and they are attractive for a number of reasons, including low production costs, the ability to assemble and modify multimeric proteins such as monoclonal antibodies (mAbs) and the ease of scalability [8].

In recent years, immunotherapy of colorectal cancer has been the subject of intensive research. Treatment of mAbs directed against tumor-associated antigen is a therapeutic strategy. As mAb appear to exert their tumoricidal effects mainly via binding to receptors of immune cells, that is by antibody-dependent cellular cytoxicity (ADCC) [9]. Lately, tumor associated colorectal cancer antigen epithelial cellular adhesion molecule (EpCAM) (also known as CO17-1A) was expressed in plant, and the recombinant plant derived antigen induced a humeral immune response in BALB/C mice [10]. The anti-EpCAM monoclonal antibody (mAb CO17-1A) recognizes the tumor associated antigen GA733 which was highly expressed in human colorectal carcinomas [11]. The mAb CO17-1A is efficacious in the treatment of micro metastasis and prevention of the recurrently of colorectal cancer in high risk patients [12]. A researcher reported that their group has recently showed that recombinant mAb can be safely purified from tobacco plant [13]. According to a previous study, that recombinant mAb CO17-1A has been expressed and assembled in tobacco plant by using the tobacco mosaic virus vector system [14]. Although such a plant virus expression system is potentially more rapid and efficient than transgenic plants, it has several drawbacks, because of transient gene expression, high mutation and large amounts of foreign genes during plant RNA viral replication [15,16]. In contrast, transgenic plants provide stable gene insertion and ease of propagation through the tissue culture of seedling [17].

The purpose of this study was to investigate whether the plant-derived monoclonal antibodies including CO17-1A (mAb^P^ CO17-1A), BR55 (mAb^P^ BR55) and plant-derived multiple monoclonal antibody CO17-1A × BR55 (multiple mAb^P^ CO17-1A × BR55) inhibit the proliferation of human colorectal cancer SW620 cells *in vivo* and *in vitro* by apoptotic pathways.

## 2. Results and Discussion

### 2.1. Results

#### 2.1.1. Obtaining Purified Plant-Derived Monoclonal Antibody (mAb^P^) CO17-1A from Transgenic Plant Leaves

**Figure 1:**
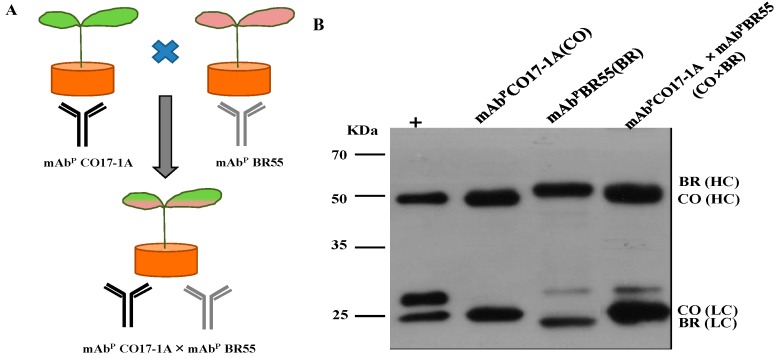
Generation of transgenic tobacco plants expressing anticancer monoclonal antibody (mAb) CO17-1A, mAb BR55 and multiple mAb CO17-1A × BR55, and its purification. (**A**) Schematic diagram of transgenic plants generation expressing multiple mAb CO17-1A × BR55 by cross-pollinate of mAb CO17-1A and mAb BR55; (**B**) Western blot of purified mammalian-derived mAb (mAb^M^) CO17-1A (40 ng), plant-derived mAb (mAb^P^) CO17-1A (40 ng), mAb^P^ BR55 and multiple mAb^P^ CO17-1A × BR55. Heavy chain (50 kDa) and were detected by anti-murine Fc conjugated HRP and Light chain (25 kDa) were detected by anti-murine F (ab') 2 conjugated horseradish peroxidase (HRP), respectively.

Tobacco transgenic plants were obtained by Agrobacterium-mediated transformation with plant expression vector carrying heavy chain (HC), and light chain (LC) of mAb CO17-1A, mAb BR55 and multiple mAb CO17-1A × BR55 (Figure 1). Transgenic Tabaco plants expressing multiple mAb CO17-1A × BR55 were generated by cross-pollinate with mAb CO17-1A and mAb BR55 (Figure 1A). Western blot confirmed the expression of both HC (50 kDa) and LC (25 kDa) of mAb^P^ CO17-1A, mAb^P^ BR55 and multiple mAb^P^ CO17-1A × BR55 in transgenic plants (Figure 1B).

#### 2.1.2. Multiple mAb^P^ CO17-1A × BR55 Inhibited the Growth of Human Colorectal Cancer SW620 Cells Treated with RAW264.7 Cells

**Figure 2:**
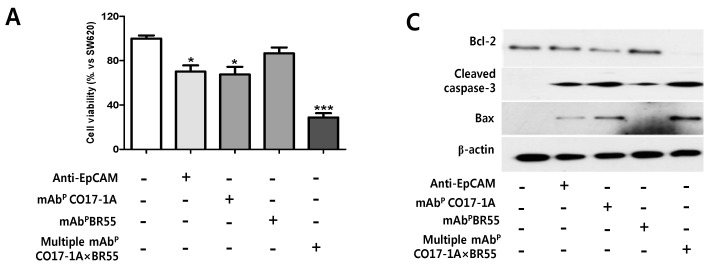

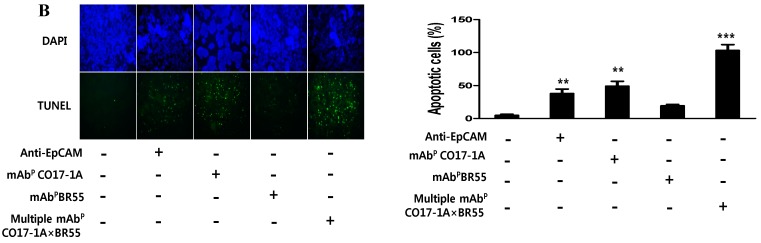
The inhibitory effect of multiple mAb^P^ CO17-1A × BR55 on cells proliferation by apoptosis in SW620 cells. (**A**) SW620 cells were seeded at 3 × 10^4^ cells/well in 96-well plates and treated with mAb^P^ CO17-1A or anti-epithelial cellular adhesion molecule (EpCAM) or multiple mAb^P^ CO17-1A × BR55, and RAW264.7 cells for 24 h. Cell proliferation was analyzed by the 3-(4,5-dimethylthiazol-2-yl)-2,5-diphenyltetrazolium bromide (MTT) assay; (**B**) Up-regulating expression of and Bax, and down-regulating expression of BCl-2. Expression of apoptosis-related proteins including cleaved caspase-3, BCl-2-associated X protein (Bax) and B-Cell lymphoma-2 (BCl-2) in SW620 cells. The expression of cleaved caspase-3, Bax, BCl-2 and β-actin proteins was measured by western blot analysis using specific antibodies; (**C**) Apoptotic cell death was determined by 4',6-diamidino-2-phenylindole (DAPI) staining and dUTP nick end labeling (TUNEL) assay. The TUNEL-positive (green) cells are an apoptotic cells (200× magnification). Apoptotic cells (DAPI-stained TUNEL-positive cells) were estimated by direct counting of fragmented nuclei after DAPI and TUNEL staining. Each band is representative of three independent experiments. Values represent mean (SD) of three experiments, each performed in triplicate. *** *p* < 0.001, ** *p* < 0.01, * *p* < 0.05 indicates a statistically significant difference from the control group.

Anticancer effects of mAb appear via binding to receptors of immune cells, which causes cancer cells death by antibody-dependent cell-mediated cytotoxicity (ADCC). To determine whether the immunoreaction of mAbs (anti-EpCAM mAb, mAb^P^ CO17-1A, mAb^P^ BR55 and multiple mAb^P^ CO17-1A × BR55) with RAW264.7 cells is inhibited to SW620 cancer cell growth, the inhibitory effect of mAbs (anti-EpCAM mAb, mAb^P^ CO17-1A, mAb^P^ BR55 and multiple mAb^P^ CO17-1A × BR55) on SW620 cancer cell growth was analyzed by 3-(4,5-dimethylthiazol-2-yl)-2,5-diphenyltetrazolium bromide (MTT) assay. Cell growth inhibition by immunoreaction of mAbs (anti-EpCAM mAb, mAb^P^ CO17-1A, mAb^P^ BR55 and multiple mAb^P^ CO17-1A × BR55) with RAW264.7 cells was also confirmed by trypan blue dye exclusion. SW620 cells viability was significantly decreased after treatments with anti-EpCAM mAb, mAb^P^ CO17-1A, and multiple mAb^P^ CO17-1A × BR55 compared to the untreated control. Moreover, treatment of SW620 cells with multiple mAb^P^ CO17-1A × BR55 (40 μM) and RAW264.7 cells resulted in significantly suppressed cell growth (Figure 2A). However, treatment with either mAb^P^ BR55 alone did not markedly inhibit growth of SW620 cancer cells (Figure 2A).

#### 2.1.3. Effect on Anti-Cancer Activity of Multiple mAb^P^ CO17-1A × BR55 in Tumor Growth of Xenograft Mouse Model

**Figure 3:**
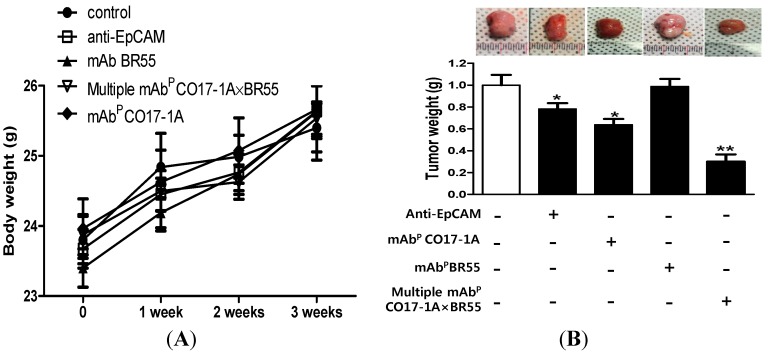
*In vivo* efficacy of multiple mAb^P^ CO17-1A × BR55 for inhibition of tumor growth in nude mice. (**A**) Body weight of mice was injected with SW620 cells and mAbs for 3 weeks; (**B**) Differential volume (size) of tumor in mAbs-treated mice; Tumor volumes (size) were recorded at 7, 14, and 21 days after injection; Tumor weight and morphology of inhibited tumor was recorded after excision. SW620 human colon cancer cells (2 × 10^6^) were injected subcutaneously (s.c.) into each mouse (thymus-deficient BALB/c nude/nude (nu/nu) mice). After tumor cell inoculation, each experimental group of 10 mice received i.p. injections of anti-EpCAM mAb, mAbP CO17-1A, mAb^P^ BR55 and multiple mAb^P^ CO17-1A × BR55, respectively (100 μg of the mAb every 2 days). ** *p* < 0.01; * *p* < 0.05 indicates a statistically significant difference from the normal group.

**Figure 4:**
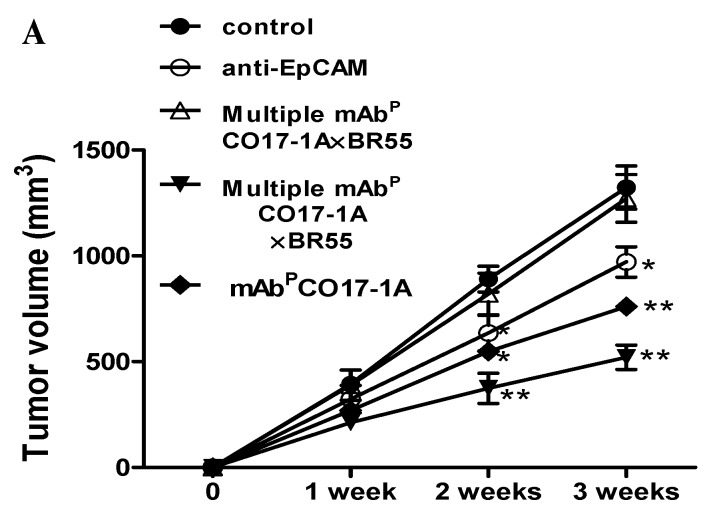

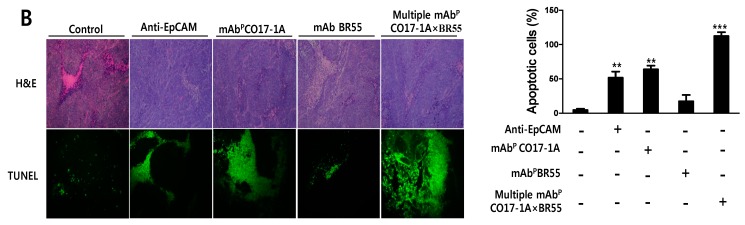
Multiple mAb^P^ CO17-1A × BR55 induces the apoptotic cell death in tumor tissues of nude mice. Apoptotic cell death was determined by hemaoxylin and eosin (H&E) staining and TUNEL assay. The TUNEL-positive (green) cells are an apoptotic cells (200× magnification). Apoptotic cells (TUNEL-positive cells) were estimated by direct counting of fragmented nuclei after TUNEL staining. Each band is representative of three independent experiments. Values represent mean (SD) of three experiments, each performed in triplicate. *** *p* < 0.001, ** *p* < 0.01, * *p* < 0.05 indicates a statistically significant difference from the control group.

**Figure 5:**
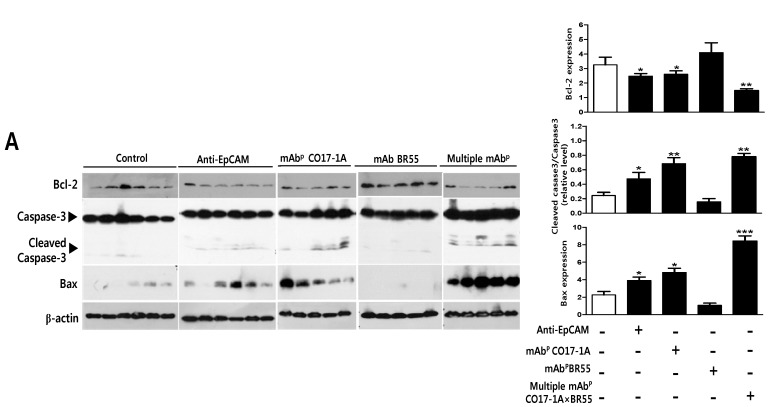

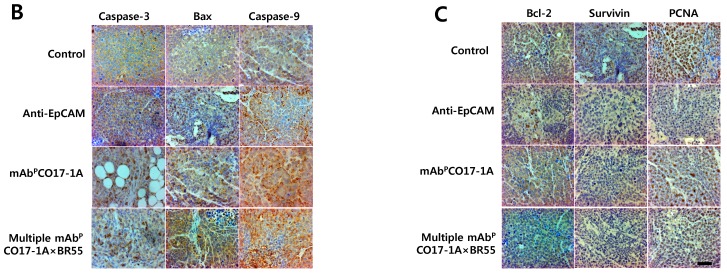
Multiple mAb^P^ CO17-1A × BR55 regulates the pro- and anti-apoptotic proteins activation in mice tumor. Apoptotic cell death was determined by immunohistochemistry, DAPI staining and TUNEL assay as described in Materials and Methods. (**A**) An equal amount of total protein (30 μg/lane) was subjected to 12% SDS-PAGE. Expression of cleaved Caspase-3, BCl-2, Bax and β-actin were detected by western blotting using specific antibodies. Here, β-actin protein was used as an internal control; (**B**) Pro-apoptotic proteins expressing cells were examined immunohistochemistry stain using the ABC kit; (**C**) Anti-apoptotic and survival proteins expressing cells analysis by Immunohistochemistry. Each band is representative of three independent experimental results. *** *p* < 0.001, ** *p* < 0.01, * *p* < 0.05 indicates a statistically significant difference from the control group. The value is the mean (SD) of three experiments, with each performed in triplicate. (**B**,**C**) Bar indicate 100 μm.

The effect of multiple mAb^P^ CO17-1A × BR55 on tumor growth was analyzed in SW620 cells-injected nude mice. No visible significant change in body weight with between control and plant-derived mAbs including anti-EpCAM mAb, mAb^P^ CO17-1A and multiple mAb^P^ CO17-1A × BR55 was observed (Figure 3A). The final tumor volume (size) was recorded at week 3, 21 days after injection of colorectal SW620 cells to mice, tumor volume was decreased in all mice groups treated respectively with anti-EpCAM mAb, mAb^P^ CO17-1A and multiple mAb^P^ CO17-1A × BR55 (Figure 3B,C). That tumor volume was significantly decreased by 55% with multiple mAb^P^ CO17-1A × BR55 treatment, by 30% with mAb^P^ CO17-1A treatment and by 15% with anti-EpCAM mAb compared with control (Figure 3B). We observed the similar results with the deceases of tumor weight, and decreases of tumor weight were higher in cell treated with multiple mAb^P^ CO17-1A × BR55 more than mAb^P^ CO17-1A and anti-EpCAM mAb (Figure 3C). Additionally, the frequency and the fluorescence intensity of dUTP nick end labeling (TUNEL)-labeled increased in tumors were treated with anti-EpCAM mAb, mAb^P^ CO17-1A and multiple mAb^P^ CO17-1A × BR55 (Figure 4A). To investigate whether the inhibition of tumor growth by plant-derived monoclonal antibodies including anti-EpCAM mAb, mAb^P^ CO17-1A and multiple mAb^P^ CO17-1A × BR55 resulted from the induction of apoptosis, we assessed the changes in apoptotic cell death by using 4',6-diamidino-2-phenylindole (DAPI) and TUNEL double staining and western blot. Apoptotic cells death was significantly increased in the plant-derived mAbs treatment compared with control (Figure 4A). Expression of anti-apoptotic protein BCl-2 was inhibited in tumor whereas the expression of pro-apoptotic proteins such as cleaved caspase-3 and Bax was increased after respective treatment with plant-derived mAb^p^, polycolonal antibody (pAb^p^) and EpCAM (Figure 4B). Moreover, treatment of mAb^P^ CO17-1A × BR55 induced higher apoptotic cell death and apoptotic regulatory protein expression than anti-EpCAM mAb and mAb^P^ CO17-1A (Figure 4). The immunohistochemical analysis of tumor sections by anti-EpCAM mAb, mAb^P^ CO17-1A and multiple mAb^P^ CO17-1A × BR55 increased the expression pro-apoptotic proteins caspase-3, Bax, and caspase-9 compared with control (Figure 5). Also, survival proteins such as survivin and PCNA were significantly decreased in the mAbs-treated tumor compared with control (Figure 5). In particular, multiple mAb^P^ CO17-1A × BR55 led to higher expression of pro-apoptotic proteins than another mAbs. These results suggested that multiple mAb^P^ CO17-1A × BR55 have the highest anticancer effects in colon cancer cell growth *in vivo*.

#### 2.1.4. Multiple mAb^P^ CO17-1A × BR55 Inhibited the Extracellular Signal Regulated Kinases 1 and 2 (ERK1/2) Phosphorylation in Colorectal Cancer SW620 Cells and Tumors

**Figure 6:**
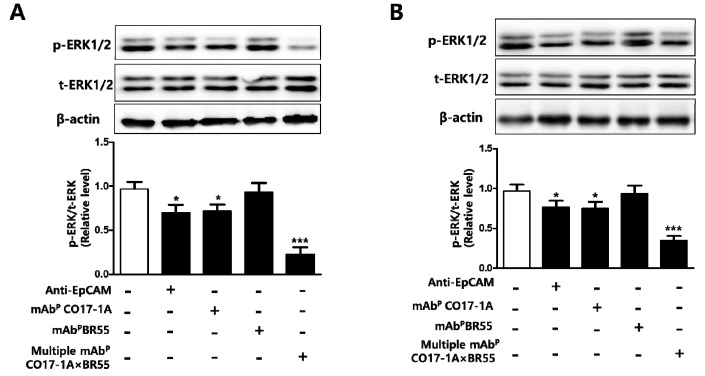
Multiple mAb^P^ CO17-1A × BR55 inhibit the extracellular signal regulated kinases 1 and 2 (ERK1/2) phosphorylation in colon cancer cells and mice tumor. Expression of beta actin, total ERK1/2 and phosphorylated ERK1/2 were detected by western blot in (**A**) SW620 colon cancer cells and (**B**) tumors. *** *p* < 0.001, * *p* < 0.05 indicates a statistically significant difference from the control group.

The inhibitory effect of multiple mAb^P^ CO17-1A × BR55 on extracellular signal regulated kinases 1 and 2 (ERK1/2) phosphorylation was analyzed in SW620 cells and tumors. Treatment of mAbs including anti-EpCAM mAb, mAb^P^ CO17-1A and multiple mAb^P^ CO17-1A × BR55 significantly suppressed the phosphorylation of ERK1/2 in SW620 colon cancer cells and tumors (Figure 6). Especially, treatment of multiple mAb^P^ CO17-1A × BR55 significantly and efficiently inhibited the ERK1/2 phosphorylation, more than other mAbs including anti-EpCAM mAb and mAb^P^ CO17-1A in SW620 colon cancer cells (Figure 6A).

### 2.2. Discussion

Our findings showed that the multiple mAb^P^ CO17-1A × BR55-mediated suppression of colon cancer cell growth *in vitro* is associated with the induction of apoptotic cell death. *In vivo* xenograft studies showed that multiple mAb^P^ CO17-1A × BR55 treatment resulted in reduced tumor weight and volume accompanied by apoptotic cell death and increased expression of pro-apoptotic cell death proteins.

Some studies reported that monoclonal antibodies against tumor associated agents have proven effective in cancer treatment, especially in conjunction with classical chemotherapy or radiotherapy [18,19]. The human colorectal carcinoma (CRC)-associated antigen CO17-1A, is highly expressed in human CRCs and is a useful passive immunotherapy target in CRC patients [20]. The glycoprotein was originally defined by anti-CO17-1A and anti-EpCAM mAbs, which bind to different epitopes on this antigen [21,22]. Previously, researchers reported the inhibition of human colorectal cancer tumor growth *in vivo* by anti-EpCAM mAb (mAb^M^ CO17-1A) [23,24]. In this study, we showed that mAb^P^ CO17-1A and multiple mAb^P^ CO17-1A × BR55 from tobacco significantly inhibited the SW620 cells and tumor growth *in vitro* and *in vivo*. We treated the mAbs and Raw264.7 cells (as an effect cells) in colorectal cancer SW620 (as a target cells) for assay of cells viability by ADCC, and find cells viability was significantly inhibited in mAbs and Raw 264.7 cells treatment. The inhibition of SW620 cells growth was occurred by anti-EpCAM mAb, mAb^P^ CO17-1A and multiple mAb^P^ CO17-1A × BR55 were able to immunobind to colorectal cancer SW620 cells. Moreover, growth inhibitory effects of multiple mAb^P^ CO17-1A × BR55 was markedly better than anti-EpCAM mAb and mAb^P^ CO17-1A. This result suggests that multiple mAb^P^ CO17-1A × BR55 inhibited colorectal cancer cell growth by immunobinding better than other mAbs. Some studies revealed that anti-colorectal cancer mAb^P^ CO17-1A and mAb^M^ CO17-1A have a similar specific binding activity on colorectal cancer cells and similar efficacy in inhibiting human colorectal tumor *in vivo* [23]. Apoptosis is one of the most prevalent pathways through which chemopreventive/chemotherapeutic agent can inhibit the overall growth of cancer cells [2]. Then, we investigated the induction of apoptosis on SW620 cells by multiple mAb^P^ CO17-1A × BR55.

Many anti-apoptotic proteins (e.g., BCl-XL, BCl-2), the TNF receptor-associated proteins 1 and 2, and pro-apoptotic proteins (e.g., caspase-3 and Bax) are regulated in variety of solid colon tumor cell [25,26]. BCl-2 family proteins play critical roles in the regulation of apoptosis by either pro-apoptotic such as Bax, or anti-apoptotic such as BCl-2. The ratio of pro- and anti-apoptotic proteins was essential to apoptosis pathway. In this study, result of western blot showed that these plant-derived mAbs decreased the expression of BCl-2, and moreover multiple mAb^P^ CO17-1A × BR55 completely inhibited the expression of BCl-2 in SW620 cells. These results suggest that multiple mAb^P^ CO17-1A × BR55 significantly and effectively induce the apoptotic cell death than anti-EpCAM mAb and mAb^P^ CO17-1A.

In contrast, this study’s results indicated that plant-derived mAbs have up-regulated the expression of cleaved caspase-3 and Bax in SW620 cells. On the one hand, we find that the tumor volume and the tumor weight were significantly decreased by plant-derived mAbs *in vivo*. On the other hand, these anti-EpCAM mAb, mAb^P^ CO17-1A and multiple mAb^P^ CO17-1A × BR55 treatments significantly decreased the expression of BCl-2, survivin and PCNA, and increased the expression of cleaved caspase-3 and Bax as determined by western blot and immunohistochemistry stain. In particular, we showed that inhibition of tumor growth, caspase-3 activation and Bax activation were more significantly up-regulated in tumor was treated with multiple mAb^P^ CO17-1A × BR55. Bax gene product dimerizes with BCl-2 and prevents it from blocking apoptosis [27]. The Bax protein controls cell death by activating caspase-3 [28]. Previous studies reported that caspases are classified based on their mode activation as either initiator or effector caspases in apoptosis [2]. Initiator caspases such caspase-8 and-9 is referred to as apical caspases, which are activated by a variety of apoptotic signals [2]. Activated or cleaved caspase-3 is crucial for induction of apoptosis as an effector [29]. These reports support our hypothesis that multiple mAb^P^ CO17-1A × BR55 significantly and effectively induce the apoptosis of colon cancer than anti-EpCAM mAb and mAb^P^ CO17-1A.

The extracellular signal regulated kinases 1 and 2 (ERK1/2) pathway which is being widely studied as a potential pharmacological target in the research of tumor target therapy [30] plays an important role in tumor initiation and progression by promoting cell survival and proliferation [31], while inhibition of ERK pathway increases sensitivity of cancer cells to apoptosis [32]. It has also been reported that ERK1/2 phosphorylation induced the suppression of caspases activation related with apoptosis in cancer cells. These reported findings support our results which suggest phosphorylation of ERK1/2 was significantly inhibited by multiple mAb^P^CO17-1A × BR55 in colon cancer [33].

## 3. Experimental Section

### 3.1. Generation of Transgenic Plants

The cDNA fragments encoding a heavy chain (HC) fused to a KDEL ER retention signal (HCK) and light chain (LC) of anti-human colorectal cancer mAb CO17-1A, anti-human breast cancer mAb BR55 and multiple mAb CO17-1A × BR55 were cloned to a pBI121 vector (Figure 1). Agrobacterium-mediated plant transformation was conducted with both the binary vectors, and transgenic lines expressing mAb CO17-1A, mAb BR55 and multiple mAb CO17-1A × BR55 were obtained as followed by a previous study [23]. Transgenic plants were selected on kanamycin (100 μg/mL) and transplanted and grown in soil.

### 3.2. Purification of Plant-Derived mAbs and Multiple mAb

To purify mAb^P^ CO17-1A, mAb^P^ BR55 and multiple mAb^P^ CO17-1A × BR55, 200 g transgenic tobacco plant leaves were homogenized with 1 extraction buffer (37.5 mM Tris/HCl pH 7.5, 50 mM NaCl, 15 mM EDTA pH 8.0, 75 mM sodium citrate, and 0.2% sodium thiosulfate) and centrifuged at 150,009 g for 30 min at 4 °C. The supernatant was filtered using a Miracloth (Calbiochem, Darmsradt, Germany) and the protein that precipitated between 16% and 40% saturation with (NH_4_)_2_SO_4_ was collected by centrifugation and the pellet was resuspended on ice in extraction buffer to one-fifth of the original volume. The final solution was then centrifuged at 250,009 g for 30 min at 4 °C. The supernatants were applied to a protein A column (GE Healthcare, Cardiff, UK), and mAb was eluted according to the manufacturer’s recommendations. After overnight dialysis against PBS (pH 7.4), mAbs were concentrated to 1 mg/mL using an Amicon Ultra spin column (GE Healthcare) with a 10 kDa cut-off and stored at 80 °C. SDS-PAGE analysis was conducted to confirm purified mAbs and multiple mAb from leaves.

### 3.3. Cell Culture

Human colorectal cancer SW620 cell line and RAW264.7 cells were purchased from Korean Cell Line Bank (KCLB, Seoul, Korea). Colorectal cancer SW620 cell line was cultured at 37 °C and 5% CO_2_ humidified air in RPMI-1640 medium, containing 10% fetal bovine serum (FBS), 100 units/mL penicillin, and 100 µg/mL streptomycin. RAW264.7 cells were also cultured at 37 °C in 5% CO_2_ humidified air, in Dulbecco’s Modified Eagle’s Medium (DMEM) with 10%FBS, 100 units/mL penicillin and 100 µg/streptomycin.

### 3.4. Cell Viability by Antibody-Dependent Cellular Cytotoxicity (ADCC)

The cell proliferation was determined by MTT assay 24 h after SW620 cells were seeded onto 24-well plates at 5 × 10^4^ cells/well and treated respectively with anti-EpCAM mAb (20 µg/mL), mAb^p^ CO17-1A (20 µg/mL), mAb^p^ BR55 (20 µg/mL), multiple mAb^p^ CO17-1A × BR55 (40 µg/mL) and RAW264.7 cells (2 × 10^5^ cells/well). At the end of the incubation period, 10 μL of (3-(4,5-dimethylthiazol-2-yl)-2,5-diphenyltetrazolium bromide (MTT; 5 mg/mL in PBS) was added each well and the plate was incubated at 37 °C for 4 h. At the end of the incubation, the culture medium was discarded and the wells were washed with PBS. Following this, 150 μL DMSO was added to each well, and the plate was incubated with shaking at room temperature. Absorbance was read at 540 nm using an ELX800 Universal Microplate Reader (Bio-Tek Instruments, Inc., Winooski, VT, USA) and subsequently the percentage (%) cell viability was calculated.

### 3.5. Antitumor Activity Study in an in Vivo Xenograft Animal Model

Six-week-old male BALB/c nude mice were purchased from Japan SLC (Hamamatsu, Japan) and were housed and maintained under sterile conditions in facilities accredited by Dams were acclimatized and kept in an isolated semi SPF barrier room with regulated temperature (23 ± 1 °C), humidity (50% ± 5%) and light/dark cycle(12/12 h). The animals were fed sterilized pellet diet by 2 M rad radiation (Samtako, Osan, Korea) and wterilized water *ad libutum*. All studies were performed in accordance with the Guid for Animal Experimentation by Wonkwang University and approved by the Institutional Animal Care and Use Committee of Wonkwang University (Approval No. WKU13-33). All efforts were made to minimize pain or discomfort of animals used. SW620 human colorectal cancer cells were injected subcutaneously (1 × 10^7^ tumor cells/0.1 mL PBS/animal) with a 27-gauge needle into the right lower flank of carrier mice. The tumor-bearing nude mice were injected intraperitoneally (i.p.) with anti-EpCAM mAb, mAb^P^ CO17-1A and multiple mAb^P^ CO17-1A × BR55 dissolved in PBS (100 μg/mouse) twice per week for 4 weeks. The weight and tumor volume of the animals were monitored twice per week. Tumor volume was measured using calipers and calculated using the formula (A × B^2^)/2, where A is the larger and B is the smaller of the two dimensions. At the end of the experiment, the animals were killed by cervical dislocation. The tumors were separated from the surrounding muscles and dermis, excised, and weighed.

### 3.6. Immunohistochemistry

All specimens were fixed in formalin and embedded in paraffin for examination. Paraffin-embedded sections were deparaffinized and rehydrated, washed in distilled water, and subjected to heat-mediated antigen retrieval treatment. Endogenous peroxidase activity was quenched by treatment with 2% hydrogen peroxide in methanol for 15 min and washing in PBS for 5 min. The sections were blocked for 30 min with 3% normal horse serum diluted in PBS; the sections were blotted and incubated with primary caspase-3, caspse-9, Bax, Bcl-2, proliferating cell nuclear antigen (PCNA), survivin and TNF-α (1:200 dilutions) at the appropriate dilution in blocking serum for 4 h at room temperature and left overnight at 4 °C. The next day, slides were washed thrice in PBS for 5 min each and incubated with biotinylated anti-mouse and rabbit antibody for 2 h. The slides were washed in PBS and incubated with the avidin–biotin–peroxidase complex (ABC, Vector Laboratories, Inc., Burlingame, CA, USA). The slides were washed and the peroxidase reaction developed with diaminobenzidine and peroxide, counterstained with hematoxylin, mounted in aqua-mount, and evaluated under a light microscope (200×; Olympus, Tokyo, Japan). A negative control was performed for every experiment by omitting the primary antibody. All slides were counterstained with hematoxylin.

### 3.7. Detection of Apoptotic Cell Death

Detection of apoptosis was done as described elsewhere method [34]. Cells were seeded in 8-chamber slides and treated, respectively, with RAW 264.7 cells and mAbs including anti-EpCAM mAb, mAb^P^ CO17-1A, mAb^P^ BR55 and multiple mAb^P^ CO17-1A × BR55 for 24 h. After, the cells were washed twice with PBS and fixed by incubation in 4% PFA for 15 minutes at room temperature. Propidium iode (PI; Sigma, St. Louis, MO, USA) staining and TdT-mediated dUTP nick and labeling (TUNEL) assays were performed using the *in situ* Cell Death Detection Kit (Roche Diagnostics, Mannheim, Germany) according to the manufacturer’s instruction.

### 3.8. Western Blot

Western blot was done as described previously by one laboratory [15]. Briefly, the SW620 colorectal cancer cells and tumor tissues were homogenized in RIPA buffer (Sigma), and then centrifuged at 13,000 rpm for 20 min at 4 °C. Proteins (30 µg/Lane) were separated on 10% SDS-PAGE gel and then transferred to nitrocellulose membrane (Hybond ECL; Amersham Pharmacia Biotech, Piscataway, NJ, USA). The blots were blocked with 5% non-fat dried milk in Tris-buffered saline for 2 h and the membrane was incubated for 16 h with a primary antibodies: BCl-2, Bax, Caspase-3, and β-actin (1:500; Santa Cruz Biotechnology, Santa Cruz, CA, USA). The blot was then incubated with the corresponding horseradish peroxidase conjugated anti-mouse, anti-rabbit secondary antibody (Santa Cruz Biotechnology), and proteins were visualized by the ECL system (Pierce, Rockford, IL, USA).

### 3.9. Statistical Analysis

All data are presented as mean (SD). Multi-group comparison was performed using one-way ANOVA and two-way ANOVA, followed by Tukey’s and Bonferroni *post hoc* pairwise comparison. A *p* value < 0.05 was considered statistically significant. All statistical analyses were performed using GraphPad Prism (Ver. 4.00; GraphPad Software Inc., La Jolla, CA, USA).

## 4. Conclusions

Therefore, this study’s results suggest that multiple mAb^P^ CO17-1A × BR55 have a significant effect on apoptosis-mediated anticancer by suppression of ERK1/2 phosphorylation in colon cancer compared to anti-EpCAM mAb and mAb^P^ CO17-1A. It is possible to reduce the production cost of the antibody used in the clinical treatment.
